# LINC00629, a KLF10-responsive lncRNA, promotes the anticancer effects of apigenin by decreasing Mcl1 stability in oral squamous cell carcinoma

**DOI:** 10.18632/aging.204396

**Published:** 2022-11-28

**Authors:** Chun Shi, Changhong Ma, Chunmei Ren, Na Li, Xiaotong Liu, Yahan Zhang, Yulong Wang, Xiaodong Li, Peng Lv, Chuanchun Han, Xiaojie Li

**Affiliations:** 1 College of Stomatology and The Second Hospital, Dalian Medical University, Dalian 116027, China; 2 Institute of Cancer Stem Cell, Dalian Medical University, Dalian 116027, China; 3 Department of Otorhinolaryngology, The First Affiliated Hospital, Dalian Medical University, Dalian 116027, China; 4 National-Local Joint Engineering Research Center for Drug-Research and Development (R&D) of Neurodegenerative Diseases, Dalian Medical University, Dalian 116044, China

**Keywords:** OSCC, apigenin, LINC00629, Mcl1, LncRNA

## Abstract

Apigenin, a naturally occurring flavonoid, is known to exhibit antitumor activity in many cancers. However, the regulatory mechanism of apigenin and the long noncoding RNAs (lncRNAs) altered upon apigenin treatment in oral squamous cell carcinoma (OSCC) remain unclear. In this study, we found that LINC00629 was significantly upregulated in response to apigenin treatment. Upregulated LINC00629 enhanced the growth-suppressive and proapoptotic effects of apigenin on OSCC cells by interacting with Mcl1 and facilitating its degradation. Subsequently, our data indicated that KLF10, an important transcription factor, directly bound to the promoter of LINC00629, facilitating its transcription and contributing to apigenin-induced LINC00629 expression. Collectively, these results suggest that the KLF10-LINC00629-Mcl1 axis plays an important role in the anticancer effects of apigenin.

## INTRODUCTION

Oral squamous cell carcinoma (OSCC) is a common and fatal head and neck cancer that accounts for more than 90% of all oral cancers worldwide and is highly prevalent in eastern nations, East Africa and South America [1]. Although multiple therapeutic strategies, such as chemotherapy, chemoradiotherapy and/or surgical resection, have been described, the overall 5-year survival rate after diagnosis remains less than 50% [2, 3]. Thus, the development of new therapeutic methods for OSCC has become particularly important.

Apigenin is a naturally occurring flavonoid and is present in many kinds of food, such as fruit, seasonings and vegetables [4, 5]. Increasing evidence has revealed that apigenin exhibits multiple documented biological activities, including anticancer activity. Apigenin has been reported to cause cell cycle arrest, suppress cell proliferation and metastatis, and induce cell death in breast, cervical, lung, colon, ovarian, skin and prostate cancers [6–11]. Some potential molecular mechanisms that mediate the anticancer effects of apigenin, including NF-κB inactivation [12], modulation of various kinase activities [13], activation of proteasomal degradation of the Her2/neu proteins [14] and suppression of PD-L1 expression, have been reported [10]. In OSCC, apigenin was found to impair cell growth and induce apoptosis. However, its potential regulatory mechanism in OSCC remains unknown.

In this study, we found that apigenin was capable of suppressing OSCC cell growth, inducing apoptosis, and inhibiting tumorigenesis by upregulating LINC00629 expression. In addition, LINC00629, as a KLF10-regulated gene, interacted with Mcl1 and resulted in Mcl1 degradation in OSCC cells. Therefore, our findings suggest that the KLF10-LINC00629-Mcl1 axis plays an important role in the anticancer effects of apigenin.

## MATERIALS AND METHODS

### Cell culture and reagents

The human OSCC cell lines UM-SCC6 and Cal-27 were cultured in DMEM containing 10% fetal bovine serum (FBS; Gibco-BRL). UM-SCC6 cells were derived from human HNC patients and were obtained from Dr. Thomas Carey at the University of Michigan. Cal-27 cells (cat.no. CC0701) were purchased from Cellcook Company (Guangzhou, China). The following antibodies and reagents were used in the study: anti-GAPDH antibody (Santa Cruz Biotechnology, Cat. no. SC-25778 1:2000), anti-Mcl1 antibody (Cell Signaling Technology, Cat. no. 94296, 1:1000), anti-Ub antibody (Santa Cruz Biotechnology, Cat. no. SC-47721, 1:1000), anti-KLF10 antibody (GeneTex, Cat. no. GTX108661, 1:200), apigenin (Cat. no. HY-N1201, MedChemExpress).

### ALDH1-positive cell detection and sphere formation assay

The ALDH1-positive population was detected using an ALDEFLUOR kit (Shanghai Stem Cell Technology Co. Ltd., Shanghai, China) following the manufacturer’s instructions. ALDH1-positive cells were detected using flow cytometric analysis (BD Accuri C6).

UM-SCC6 cells with or without LINC00629 knockdown were treated with 20 μM apigenin. Spheres were enriched from the cells by culturing 3000 cells/mL in serum-free DMEM-F12 medium (Gibco) supplemented with B27 (1:50, Invitrogen) and 20 ng/mL EGF and bFGF. Cells were cultured for 2 weeks, and the spheres were counted.

### Cell viability and colony formation assays

The proliferation of OSCC cells was determined by an MTT assay. In brief, cells were seeded at a density of 2000 cells/well in 96-well plates and incubated overnight. The next day, the cells were treated with apigenin or DMSO as the control at the indicated concentrations for 36 h. To examine cell proliferation, 20 μL of MTT solution (5 mg/ml) was added to each well. The absorbance was measured at 570 nm and 630 nm with a spectrometer.

For the colony formation assay, UM-SCC6 and Cal-27 cells treated with apigenin as indicated were diluted with a single-cell suspension, and 1000 cells were cultured in every well of 6-well plate at 37° C in a 5% CO_2_ incubator for 2 weeks. Then, the colonies were stained with 0.04% crystal violet- 2% ethanol and counted.

### Flow cytometric analysis

Apoptosis was detected using a Cell Apoptosis Analysis Kit (Yeasen, China). Briefly, OSCC cells were treated with DMSO (control) or apigenin for 36 h. Then, the samples were subjected to Annexin V and PI double-staining with an apoptosis assay kit (Yeasen, China) according to the manufacturer’s instructions.

### qRT–PCR and RT–PCR

Total RNA was isolated using TRIzol (Invitrogen). One microgram of total RNA was used to synthesize cDNA using the PrimeScriptTM RT reagent kit (Takara, RR047A) according to the manufacturer’s instructions. The primers used are shown in Supplementary Table 1.

### ChIP assay

Cells were crosslinked with 1% formaldehyde for 10 min at room temperature. The ChIP assay was performed according to the manufacturer’s instructions using the anti-KLF10 antibody and a kit (Millipore, Merck KGaA, Darmstadt, Germany). Anti-rabbit IgG was used as the control. The bound DNA fragments were eluted and amplified by PCR. PCR products were separated by gel electrophoresis.

### RNA sequencing analysis and label-free quantitative proteomics

UM-SCC6 cells were treated with or without 40μM apigenin for 36h. Then the cells were collected and transported to BioMaker. RNA extraction, library construction, sequencing and data analysis were performed by BioMaker, Beijing, China.

For label-free quantitative proteomics, 10^6^ UM-SCC6 cells with or without LINC00629 knockdown were collected and transported to Jingjie PTM Biolab, Hongzhou, China.

### Protein half-life assay and *in vivo* Mcl1 ubiquitylation assay

For the Mcl1 half-life assay, OSCC cells with or without LINC00629 depletion were pretreated with 40 μM apigenin for 33 h and then treated with CHX (Sigma, 10 mg/ml) for the indicated durations before collection and Western blot analysis.

For the Mcl1 ubiquitylation assay, HA-ubiquitin was transfected into UM-SCC6 and Cal-27 cells with or without LINC00629 depletion. The cells were then treated with 20 μM MG132 (Calbiochem) for 8 h. These cells were lysed with NP40 lysis buffer and incubated with the indicated primary antibodies. After washing with PBS three times, proteins were released from the beads by boiling in SDS–PAGE sample buffer and analyzed using the anti-Ub antibody.

### RNA pulldown assay

RNA pulldown assays were performed as previously described [15]. Briefly, OSCC cell lysates were prepared by ultrasonication in RIP buffer (150 mM KCl, 25 mM Tris (pH 7.4), 0.5 mM dithiothreitol, 0.5% NP-40, complete protease inhibitors cocktail and RNase inhibitors) and pre-cleared against streptavidin magnetic beads. *In vitro* transcribed RNA adsorbed to streptavidin magnetic beads were then incubated with cell lysate at 4° C for 4 h before washing five times in RIP buffer and elution in Laemmli sample buffer. Eluted proteins were separated by SDS–PAGE for mass spectrometry or Western blot analysis.

### RNA interference and virus infection

To generate lentiviral shRNA constructs targeting human LINC00629, the LINC00629 sequence was cloned into the pLKO.1-puro vector. The target sequence LINC00629 No.1 5- CGTGAGTTTATAAGCGGAT-3; No.2 5- GGGTTGTAGTAGGTGTATA-3. The siRNAs for KLF10, KLF14 and EGR1 were purchased from Sigma: KLF10 siRNA SASI_Hs01_00231862 and SASI_Hs01_00231863; KLF14 siRNA SASI_Hs01_00168202 and SASI_Hs02_00363787; EGR1 siRNA SASI_Hs01_00232227 and SASI_Hs01_00232228. The LINC00629 siRNA pool was purchased from Horizon.

### Promoter reporters and dual-luciferase assay

The promoter of LINC00629 and the matching mutant were constructed into a pGL3-basic vector. Luciferase activity was measured in a 1.5-ml Eppendorf tube with a Promega Dual-Luciferases Reporter Assay kit (Promega E1980) according to the manufacturer’s protocol after transfection. Relative Renilla luciferase activity was normalized to firefly luciferase activity.

### Statistics and data analyses

The data are expressed as the means ± SDs and were statistically evaluated using GraphPad Prism 5 and 7. Data are presented as the means ± SDs. Multiple comparisons between treatment groups and controls were performed using Dunnett’s least significant difference (LSD) test. Values of p <0.05 were considered statistically significant.

### Availability of data and materials

Data sharing is not applicable to this article.

## RESULTS

### Apigenin promotes LINC00629 expression in OSCC cells

**Figure 1:**
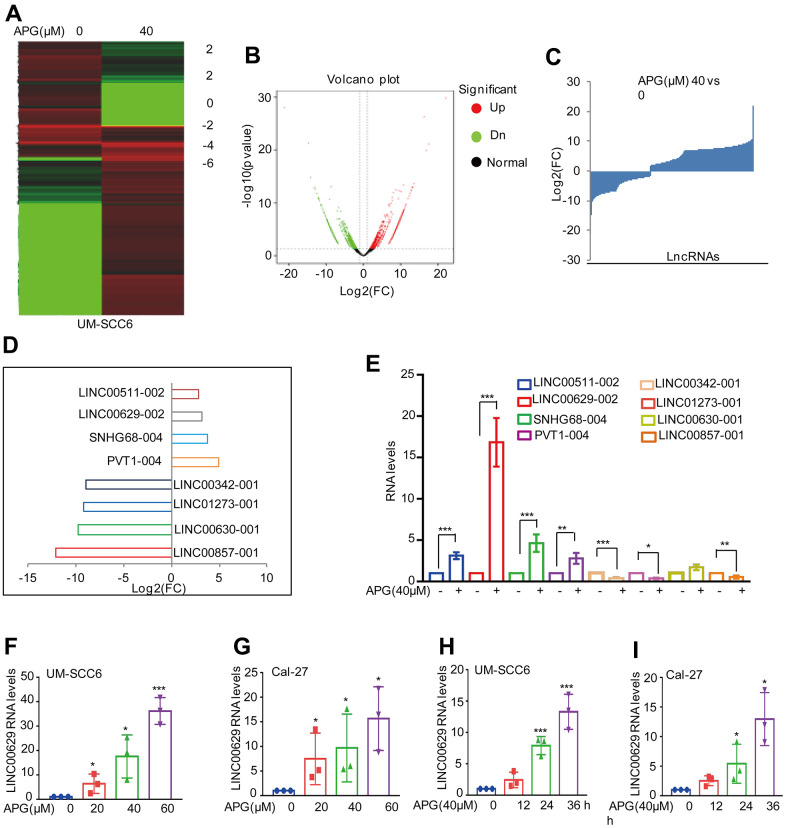
**Apigenin induced LINC00629 expression.** (**A**–**C**) UM-SCC6 cells were treated with or without apigenin and then subjected to RNA sequencing analysis. The altered lncRNAs are listed. (**D**) Eight altered lncRNAs were selected and are listed. (**E**) The expression of these lncRNAs was confirmed by qRT–PCR. (**F**, **G**) UM-SCC6 and Cal-27 cells were treated with apigenin at different concentrations, and LINC00629 expression was analyzed by qRT–PCR. (**H**, **I**) UM-SCC6 and Cal-27 cells were treated with 40 μM LINC00629 for the indicated times, and the expression level of LINC00629 was analyzed by qRT–PCR. In (**E**–**I**), the results represent three independent experiments; *p<0.05, **p<0.01, ***p<0.001.

To determine anticancer effects of apigenin on OSCC cells, we first examined cell proliferation and apoptosis under apigenin treatment and found that apigenin significantly decreased cell viability and inhibited cell proliferation in a concentration-dependent manner (Supplementary Figure 1A–1C). The subsequent flow cytometric analysis showed that the numbers of apoptotic cells upon apigenin treatment were markedly increased (Supplementary Figure 1D, 1E). To further investigate the anticancer molecular mechanism, we treated UM-SCC6 cells with or without 40 μM apigenin and the lncRNA profiles were analyzed. As shown in Figure 1A–1C, 1655 upregulated and 963 downregulated lncRNAs were identified (Supplementary Table 2). From the changed lncRNAs triggered by apigenin, eight significantly altered lncRNAs—LINC00857-001, LINC00630-001, LINC01273-001, LINC00324, PVT1-004, SNHG68-004, LINC00629-002 and LINC00511-002—were selected (Figure 1D). The alterations in these lncRNAs were confirmed by qRT–PCR. We found that LINC00629-002 was dramatically increased in response to apigenin treatment (Figure 1E). Thus, we chose LINC00629-002, which was named LINC00629, for subsequent functional studies.

To further determine whether the increase in LINC00629 was dependent on the concentration of apigenin. UM-SCC6 and Cal-27 cells were treated with different concentrations of apigenin for 36 h, and the LINC00629 expression level was determined. Our data indicated that the expression of LINC00629 was upregulated with increasing concentrations of apigenin (Figure 1F, 1G). Similarly, LINC00629 was also gradually upregulated with increasing treatment time (Figure 1H, 1I).

### LINC00629 facilitates the anticancer effects of apigenin in OSCC cells

**Figure 2:**
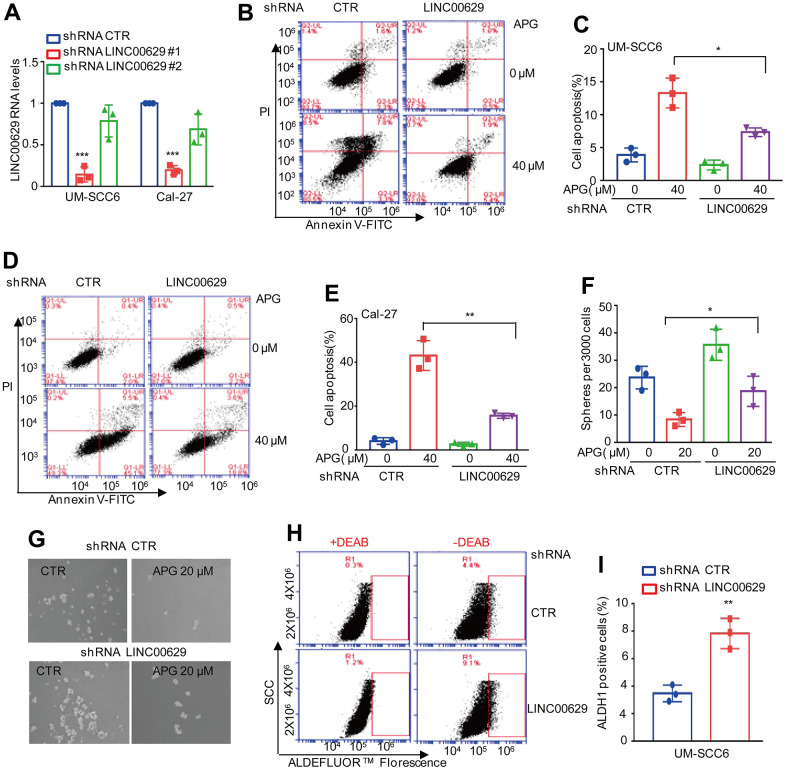
**LINC00629 knockdown impaired the anticancer effects of apigenin.** (**A**) LINC00629 was knocked down in UM-SCC6 and Cal-27 cells, and the expression of LINC00629 was analyzed by qRT–PCR. (**B**–**E**) UM-SCC6 and Cal-27 cells with or without LINC00629 knockdown were treated with apigenin as indicated. Apoptosis was analyzed by flow cytometry. (**F**, **G**) UM-SCC6 cells with or without LINC00629 knockdown were treated with 20 μM apigenin for 36 h. The mammosphere-forming abilities were analyzed. The column charts on the right show the quantitation of spheres. (**H**, **I**) The percentage of ALDH1-positive cells was determined in UM-SCC6 cells with or without LINC00629 knockdown. In (**A**, **C**, **E**, **F**, **I**) the results represent three independent experiments; *p<0.05, **p<0.01, ***p<0.001.

To assess the biological role of LINC00629 in apigenin-induced tumor suppression, we first stably knocked down LINC00629 in UM-SCC6 and Cal-27 cells. The knockdown efficiency was demonstrated by qRT–PCR. As shown in Figure 2A, the inhibitory effect of the first shRNA was stronger than that of another shRNA. Thus, we used the first one in the subsequent studies. These cells with or without LINC00629 knockdown were treated with apigenin as indicated. Apoptosis was analyzed. As shown in Figure 2B–2E, depletion of LINC00629 decreased apigenin-induced apoptosis. To exclude off-target effects, we also used siRNAs to knock down LINC00629 in UM-SCC6 cells, and apoptosis was then analyzed. Similarly, knockdown of LINC00629 decreased apigenin-induced apoptosis (Supplementary Figure 2A–2C). Additionally, sphere formation and ALDH1-positive sorting assays were performed to investigate the effects of LINC00629 on OSCC stem cells. Our data indicated that inhibition of LINC00629 impaired the apigenin-induced OSCC stem cell decrease and increased the number of ALDH1-positive cells (Figure 2F–2I). Collectively, these results suggest that LINC00629 plays an important role in the anticancer effects of apigenin.

### LINC00629 contributes to apigenin-induced Mcl1 downregulation

**Figure 3:**
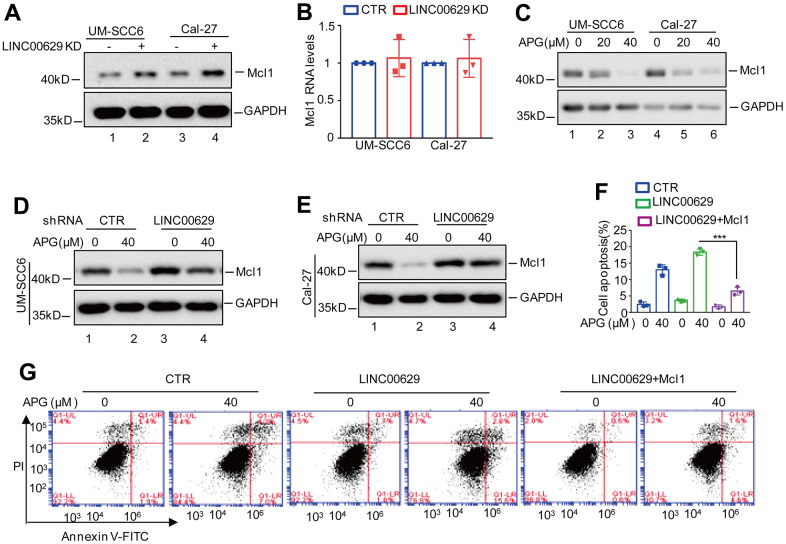
**LINC00629 facilitated apigenin-induced Mcl1 decrease.** (**A**, **B**) UM-SCC6 and Cal-27 cells with or without LINC00629 knockdown were harvested. The protein and mRNA level of Mcl1 were analyzed by Western blotting and qRT–PCR. (**C**) UM-SCC6 and Cal-27 cells were treated with apigenin at 0 μM or 20 μM for 36 h. The protein level of Mcl1 was determined by Western blotting. (**D**, **E**) UM-SCC6 and Cal-27 cells with or without LINC00629 knockdown were treated with or without 40 μM apigenin for 36 h. The protein level of Mcl1 was analyzed by Western blotting. (**F**, **G**) Mcl1 was transfected into UM-SCC6 cells with or without LINC00629 overexpression, and the cells were then treated with apigenin as indicated. Apoptosis was analyzed by flow cytometry. In (**F**) the results represent three independent experiments; ***p<0.001.

To uncover the molecular mechanism whereby LINC00629 contributed to the antitumor effects of apigenin, we used label-free quantitative proteomics to identify the differentially expressed proteins in UM-SCC6 cells with or without LINC00629 knockdown. We found that depletion of LINC00629 upregulated Mcl1 expression (Supplementary Figure 3A–3C). To confirm this, we determined the expression levels of Mcl1 in OSCC cells with or without LINC00629 knockdown. We found that inhibition of LINC00629 elevated Mcl1 protein levels but did not alter the Mcl1 mRNA level (Figure 3A, 3B). Additionally, we also found that the protein level of Mcl1 was significantly decreased in response to apigenin treatment, and the reduction was abolished by LINC00629 knockdown (Figure 3C–3E). Subsequently, we wanted to determine whether LINC00629 promoted apigenin-induced apoptosis by regulating Mcl1 expression. To prove this, we transfected Mcl1 into UM-SCC6 cells with or without LINC00629 overexpression. The expression levels of LINC00629 and Mcl1 were analyzed (Supplementary Figure 3D, 3E). Then cell apoptosis was detected. As shown in Figure 3F, 3G, we found that overexpression of Mcl1 abolished the apigenin-induced increase in apoptosis in LINC00629-overexpressing cells. Taken together, our data indicate that LINC00629 promotes the apigenin-induced decrease in the Mcl1 protein level and enhances the anticancer ability of apigenin by regulating Mcl1 expression.

### LINC00629 interacts with Mcl1 and promotes its degradation

**Figure 4:**
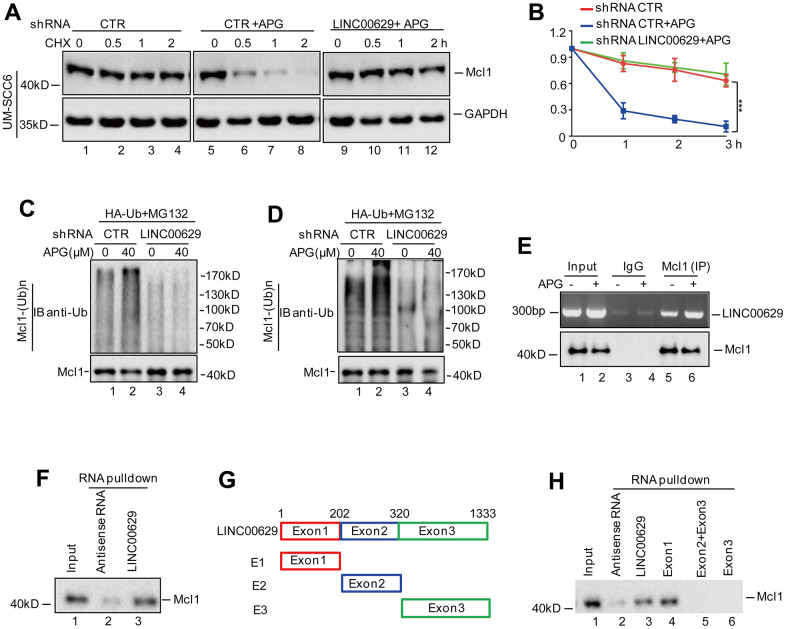
**LINC00629 interacted with Mcl1.** (**A**, **B**) UM-SCC6 cells with or without LINC00629 knockdown were pretreated with 40 μM for 33 h and were then treated with 10 μM CHX for the indicated times. The expression level of Mcl1 was determined by Western blotting. The results represent three independent experiments; ***p<0.001. (**C**, **D**) HA-Ub was transfected into Cal-27 and UM-SCC6 cells with or without LINC00629 knockdown. The cells were treated with MG132 for 8 h before collection. The whole-cell lysate was subjected to immunoprecipitation with an anti-Mcl1 antibody and Western blotting with an anti-HA antibody to detect ubiquitylated Mcl1. (**E**) The anti-Mcl1 antibody was used to coprecipitate LINC00629 in whole-cell lysates of UM-SCC6 cells treated with or without apigenin. (**F**) Biotin-labeled LINC00629 or antisense RNA was pulled down with Mcl1 in whole-cell lysates of UM-SCC6 cells. (**G**) Schematic illustration of the division of LINC00629 into three fragments corresponding to individual exons of the LINC00629 gene (E1, E2 and E3) along with the corresponding truncated bodies used. (**H**) Biotin-labeled LINC00629, truncated bodies or antisense RNA were pulled down with Mcl1 in whole-cell lysates of UM-SCC6 cells.

It is well known that proteasome-dependent degradation is one of the main modalities controlling the Mcl1 abundance [16]. To confirm whether LINC00629 affects Mcl1 expression in a proteasome-dependent manner, we first detected the effect of LINC00629 on the stability of Mcl1 under apigenin treatment. We found that apigenin reduced the stability of Mcl1. However, the decrease was abolished by LINC00629 knockdown (Figure 4A, 4B). Except that, the proteasome inhibitor MG132 also could abolished the downregulation of Mcl1 induced by LINC00629 (Supplementary Figure 3F). Consistent with these findings, depletion of LINC00629 diminished apigenin-induced ubiquitination of Mcl1 in OSCC cells (Figure 4C, 4D).

Considering that LINC00629 facilitated Mcl1 degradation, we wanted to determine whether LINC00629 interacts with Mcl1. To test this hypothesis, an RNA immunoprecipitation (RIP) assay was performed. As shown in Figure 4E, LINC00629 was enriched by the anti-Mcl1 antibody relative to IgG, and the enrichment of LINC00629 in the Mcl1 immunoprecipitate was increased by apigenin. In addition, we carried out an RNA pulldown assay in which biotin-labeled LINC00629 and antisense RNA were synthesized *in vitro* and incubated with whole-cell lysates of UM-SCC6 cells. As expected, Mcl1 was precipitated by LINC00629 (Figure 4F).

To identify the key regions in LINC00629 that are required for its interaction with Mcl1, we performed deletion mapping experiments. We observed that the region from nucleotides 1 to 202 was mainly responsible for binding to Mcl1 (Figure 4G, 4H) and the region also reduced the expression of Mcl1 which was consistent with the full length of LINC00629 (Supplementary Figure 3G). Collectively, these results indicate that LINC00629 could accelerate Mcl1 degradation via their interaction.

### KLF10 promotes LINC00629 expression in response to apigenin treatment

**Figure 5:**
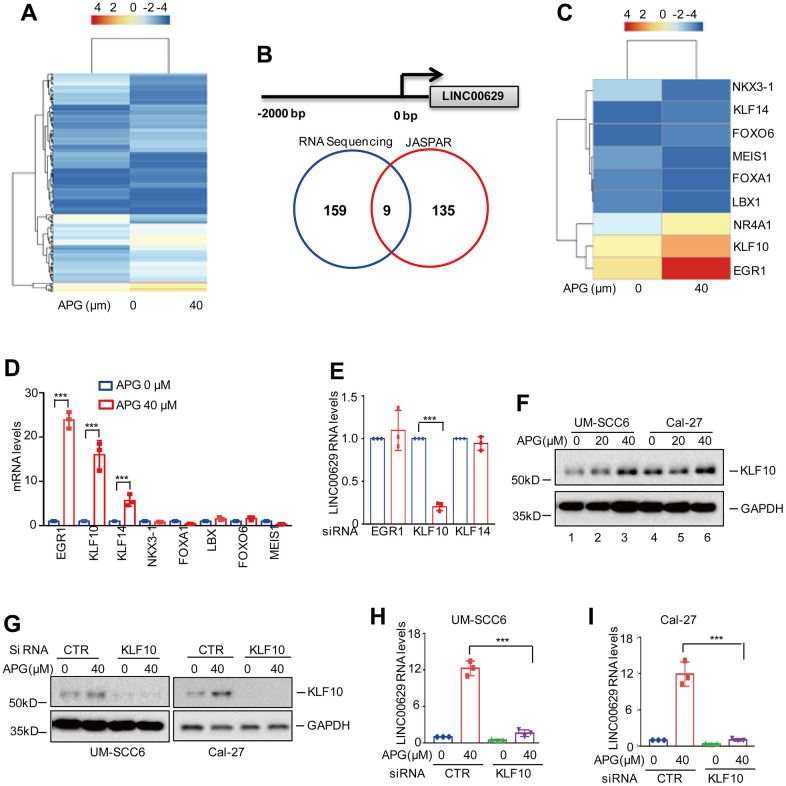
**KLF10 upregulated LINC00629 expression.** (**A**) The altered transcription factors under apigenin treatment in UM-SCC6 cells were selected from the RNA sequencing data and are listed. (**B**) Overlaps indicating the numbers of transcription factors between RNA sequencing analysis and JASPAR prediction. (**C**) Heatmap showing the 9 overlapping transcription factors. (**D**) UM-SCC6 cells were treated with or without apigenin as indicated. The RNA levels of transcription factors were analyzed. (**E**) EGR1, KLF10 and KLF14 were knocked down using siRNAs. The expression level of LINC00629 was analyzed by qRT–PCR. (**F**) UM-SCC6 and Cal-27 cells were treated with apigenin as indicated, and the expression level of KLF10 was determined by Western blotting. (**G**) UM-SCC6 and Cal-27 cells with or without KLF10 knockdown were treated with apigenin as indicated for 36 h. The protein level of KLF10 was determined by Western blotting. (**H**, **I**) The expression level of LINC00629 was analyzed by qRT–PCR. In (**D**, **E**, **H**, **I**) the results represent three independent experiments; *p<0.05, **p<0.01, ***p<0.001.

To uncover the mechanism by which apigenin induced LINC00629 expression, we first searched the transcription factors altered under apigenin treatment from the RNA sequencing data in Figure 3 and found 168 significantly altered transcription factors (Figure 5A and Supplementary Table 3). Then, we inspected the upstream sequence of LINC00629 using JASPAR software and identified 144 candidate transcription factors (Supplementary Table 4). Interestingly, we found 9 overlapping transcription factors (Figure 5B, 5C). To further determine which of these is the actual transcription factor of LINC00629, we confirmed transcription factor expression and found that EGR1, KLF10 and KLF14 were significantly upregulated under apigenin treatment, which was consistent with the RNA sequencing data (Figure 5D). Subsequently, we knocked down EGR1, KLF10 and KLF14 using siRNAs in UM-SCC6 cells and checked LINC00629 expression by qRT-PCR analysis. As shown in Figure 5E, knockdown of KLF10 but not EGR1 or KLF14 observably inhibited LINC00629 expression, indicating that KLF10 may be a potential transcription factor of LINC00629. To further prove this, we determined the protein level of KLF10 under apigenin treatment. Consistent with the mRNA level, the protein level of KLF10 was significantly increased after apigenin treatment (Figure 5F). Depletion of KLF10 abolished apigenin-induced LINC00629 upregulation in OSCC cells (Figure 5G–5I).

### KLF10 directly binds to the promoter of LINC00629

**Figure 6:**
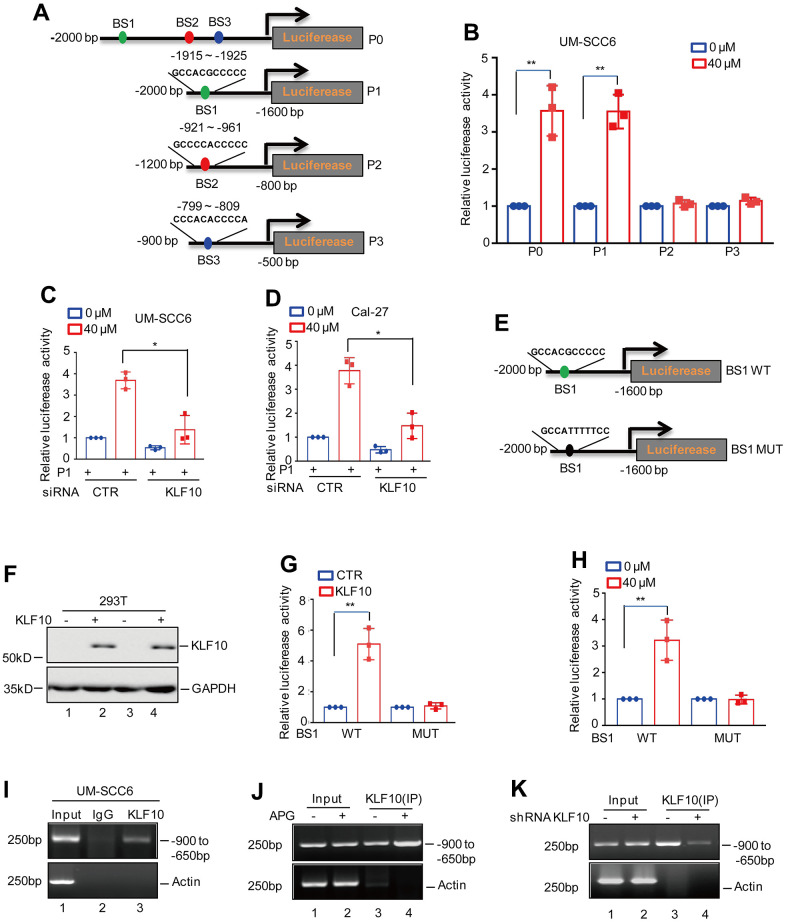
**KLF10 directly bound to the promoter of LINC00629.** (**A**) Schematic illustration of the pGL3-based reporter constructs (P0-P3) used in luciferase assays to examine the transcriptional activity of LINC00629. (**B**) The promoters of LINC00629, named P0-P3, were individually transfected into UM-SCC6 cells, and the cells were treated with or without 40 μM apigenin. Luciferase activity was measured. (**C**, **D**) The promoter of P1 was transfected into UM-SCC6 and Cal-27 cells with or without KLF10 knockdown, and the cells were then treated with or without 40 μM apigenin for 36 h. Luciferase activity was measured. (**E**) Schematic illustration of the KLF10 wild-type binding site (BS1 WT) and the matching mutant (BS1 MUT) that were used in luciferase assays. (**F**, **G**) BS1 WT and MUT were transfected into 293T cells with or without KLF10 overexpression. KLF10 expression was determined by Western blotting (**F**). Luciferase activity was detected (**G**). (**H**) BS1 WT and BS1 MUT were transfected into UM-SCC6 cells treated with or without apigenin. The luciferase activity was measured. (**I**–**K**) ChIP assay showing the binding of KLF10 to the promoter of LINC00629 in UM-SCC6 cells with or without apigenin treatment or KLF10 knockdown. Isotype-matched IgG was used as the negative control. In (**B**–**D**, **G**, **H**) the results represent three independent experiments; *p<0.05, **p<0.01, ***p<0.001.

To further prove that KLF10 transcriptionally upregulates LINC00629, we investigated the effect of KLF10 on the promoter activity of LINC00629. Based on the analysis with JASPAR software, three potential binding sites were identified. To verify this hypothesis, we first cloned the upstream sequence of LINC00629 and different truncations by PCR. We inserted these sequences into pGL3-based luciferase reporter plasmids, which were named P0-P3, and then transfected the plasmids into UM-SCC6 cells treated with or without apigenin (Figure 6A). As shown in Figure 6B, the luciferase activities of P0 and P1 were increased in response to apigenin treatment. However, the increase disappeared when P2 and P3 were transfected, suggesting that the P1 region was essential for apigenin-induced LINC00629 expression. To further confirm this hypothesis, we transfected P1 into OSCC cells with or without KLF10 knockdown. As shown in Figure 6B, 6C, loss of KLF10 suppressed the apigenin-induced increase in luciferase activity. Additionally, we constructed two pGL3-based luciferase reporter plasmids containing BS1 WT and BS1Mut (Figure 6E). These plasmids were individually transfected into 293T cells with or without KLF10 overexpression. The expression level of KLF10 was determined by Western blotting (Figure 6F). The luciferase activities of the WT and Mut BS1 were measured. We found that the activity of BS1 but not the mut was significantly increased in response to KLF10 overexpression (Figure 6G). Similar results were obtained in UM-SCC6 cells, in which apigenin dramatically elevated the luciferase activity of BS1 but not that of mut (Figure 6H). These results indicated that BS1 is a positive KLF10-binding site in the LINC00629 promoter.

Furthermore, the following chromatin immunoprecipitation (ChIP) assays showed that the chromatin fragment containing BS1 was specifically present in anti-KLF10 immunoprecipitation (Figure 6I). The binding capacity of KLF10 to the LINC00629 promoter was enhanced under apigenin treatment and was weakened by KLF10 knockdown (Figure 6J, 6K). Taken together, these findings indicate that the BS1 region is of great significance for KLF10 to elevate LINC00629 expression.

### KLF10 enhances the anticancer effects of apigenin and decreases Mcl1 expression by upregulating LINC00629 expression

**Figure 7:**
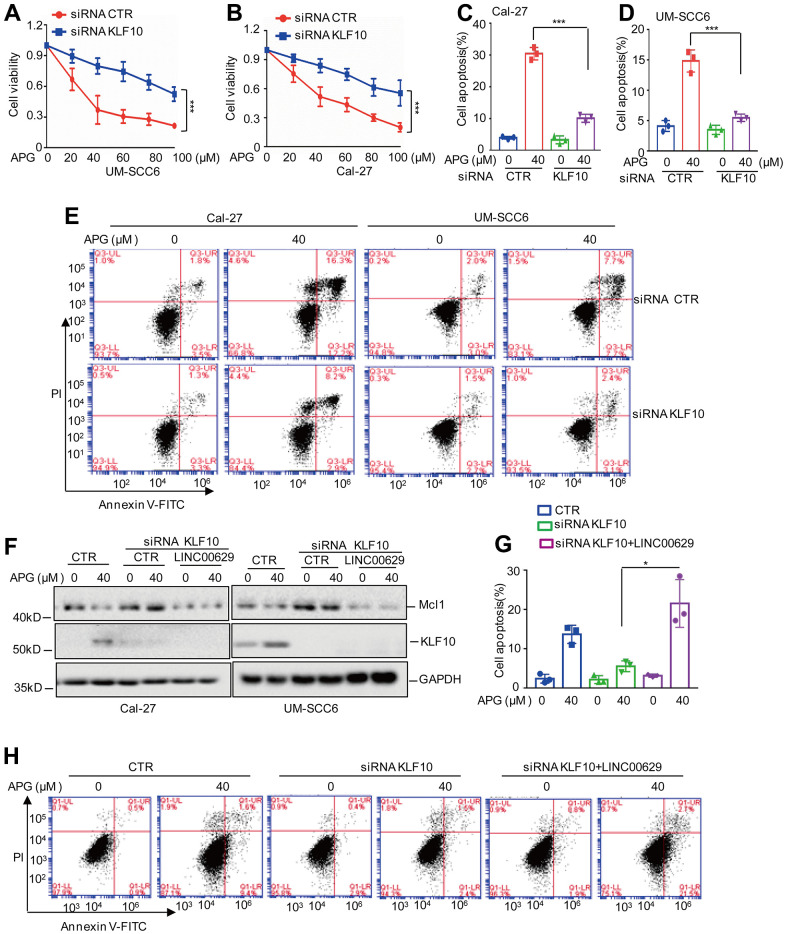
**The KLF10-LINC00629-Mcl1 axis plays an important role in the anticancer activity of apigenin.** (**A**, **B**) UM-SCC6 and Cal-27 cells with or without KLF10 knockdown were treated with apigenin as indicated. Cell viability was measured by an MTT assay. (**C**–**E**) Apoptosis was analyzed by flow cytometry. (**F**) LINC00629 was overexpressed in Cal-27 and UM-SCC6 cells with or without KLF10 knockdown, and the cells were treated with 40 μM apigenin for 36 h. The expression levels of Mcl1 and KLF10 were determined by Western blotting. (**G**, **H**) Apoptosis was analyzed by flow cytometry. In (**A**–**D**, **G**) the results represent three independent experiments; *p<0.05, **p<0.01, ***p<0.001.

To determine whether KLF10 enhances the anticancer effects of apigenin and downregulates Mcl1 expression by regulating LINC00629, we first assessed the role of KLF10 in the anticancer activity of apigenin and found that knockdown of KLF10 increased OSCC cell viability and decreased apoptosis in response to apigenin treatment (Figure 7A–7E). Subsequently, we knocked down KLF10 in OSCC cells with or without LINC00629 overexpression, and these cells were then treated with apigenin. Mcl1 expression and apoptosis were analyzed by Western blotting and flow cytometry. Our data indicated that inhibition of KLF10 blocked the apigenin-induced decrease in the Mcl1 protein level and increase in apoptosis. However, the phenotype was abolished when LINC00629 was overexpressed (Figure 7F–7H). Therefore, these results indicate that the role of KLF10 in the anticancer effects of apigenin is dependent on upregulation of LINC00629 expression.

## DISCUSSION

Although numerous therapeutic strategies are emerging, OSCC remains a major life-threatening malignancy. Unfortunately, patients with advanced OSCC usually have a low survival rate owing to resistance to traditional therapeutic agents. Thus, it is of great urgency to explore high-efficiency and low-toxicity drugs for OSCC treatment.

Apigenin is a naturally occurring flavonoid that is present in many kinds of food, such as fruits, seasonings and vegetables [13]. Accumulating evidence has indicated that apigenin exhibits antitumor activity in many cancers; for example, apigenin inhibits cell proliferation, migration, and invasion by targeting Akt in the human lung cancer cell line A549 [17]. Apigetrin inhibits gastric cancer progression by inducing apoptosis and regulating the ROS-modulated STAT3/JAK2 pathway [18]. In OSCC, apigenin was also reported to inhibit cell growth and induce apoptosis. However, the potential molecular mechanism remains unknown. In this study, we observed that the level of the lncRNA LINC00629 was significantly increased under apigenin treatment and that the increased LINC00629 contributed to the anticancer activity of apigenin.

LINC00629 is a long intergenic noncoding RNA mapped to chromosome X (Xq26). Recently, LINC00629 was reported to decrease the migration and invasion of JEG-3 cells [19]. In gastric cancer, LINC00629 was found to suppress tumor progression by upregulating AQP4 and competitively binding to miR-196b-5p [20]. In accordance with previous evidence, we found that LINC00629, as a tumor suppressor, promoted apigenin-induced apoptosis in OSCC. It has been recognized that LINC0629 can act as a miR-196b-5p sponge to inhibit gastric cancer progression [20]. However, we found that LINC02629 can interact with Mcl1 and facilitate Mcl1 degradation, which enhanced the anticancer effect of apigenin in OSCC.

Dysregulation of lncRNA expression at the transcriptional level has been frequently reported in various cancers [21, 22]. Many important transcription factors have been indicated to regulate lncRNA expression in cancer [23, 24]. To uncover the mechanism by which apigenin increases the level of LINC00629 in OSCC cells, we screened the altered transcription factors and found that KLF10 can bind to the promoter region of LINC00629, leading to the apigenin-induced LINC00629 increase.

KLF10, also called TGFβ inducible early gene-1 (TIEG1), is a member of the Krüppel-like family of transcription factors [25, 26]. Previous studies indicated that KLF10 is a key tumor suppressor gene in multiple cancers [27]. However, the role of KLF10 in OSCC is still unknown. In this study, we found that the mRNA level of KLF10 was significantly increased upon apigenin treatment, which enhanced LINC00629 expression in OSCC. However, the molecular mechanism by which KLF10 expression is increased by apigenin has not been fully elucidated. Thus, the underlying mechanism will be explored in further studies.

## CONCLUSIONS

In summary, our data indicated that LINC00629, as a KLF10-regulated gene, promoted Mcl1 degradation, which enhanced the anticancer activity of apigenin. Thus, our findings suggest that the KLF10-LINC00629-Mcl1 axis plays an important role in the anticancer effects of apigenin.

## Supplementary Material

Supplementary Figures

Supplementary Table 1

Supplementary Table 2

Supplementary Table 3

Supplementary Table 4
