# Supplementary material for: LINC00629, a KLF10-responsive lncRNA, promotes the anticancer effects of apigenin by decreasing Mcl1 stability in oral squamous cell carcinoma
Source: Aging (Albany NY). 2022 Nov 28;14(22):9149–66. doi: 10.18632/aging.204396 (PMC9740369; doi:10.18632/aging.204396)
Supplement: Supplementary Figures [file aging-14-204396-s001.pdf]

SUPPLEMENTARY FIGURES

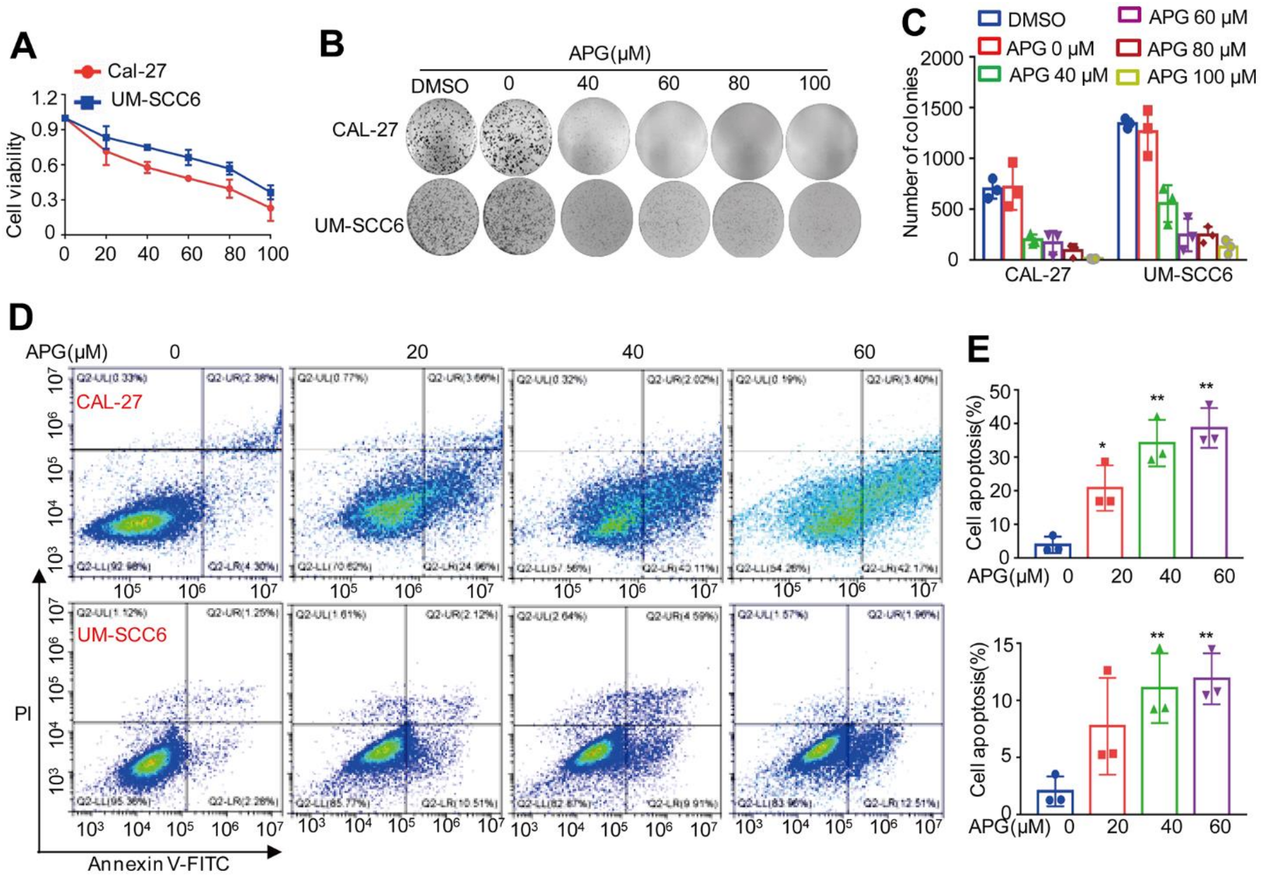

Supplementary Figure 1. (**A**) Cal-27 and UM-SCC6 cells were treated with apigenin (APG) at various concentrations for 36 h. Cell viability was measured by an MTT assay. (**B**, **C**) Cal-27 and UM-SCC6 cells were treated with apigenin at the indicated concentrations. Cell proliferation was analyzed by a colony formation assay. Cell colonies were counted. (**D**, **E**) Cal-27 and UM-SCC6 cells were treated with apigenin as indicated for 36 h, and apoptosis was then analyzed. In (**C**, **E**) the results represent three independent experiments; *p<0.05, **p<0.01, ***p<0.001.

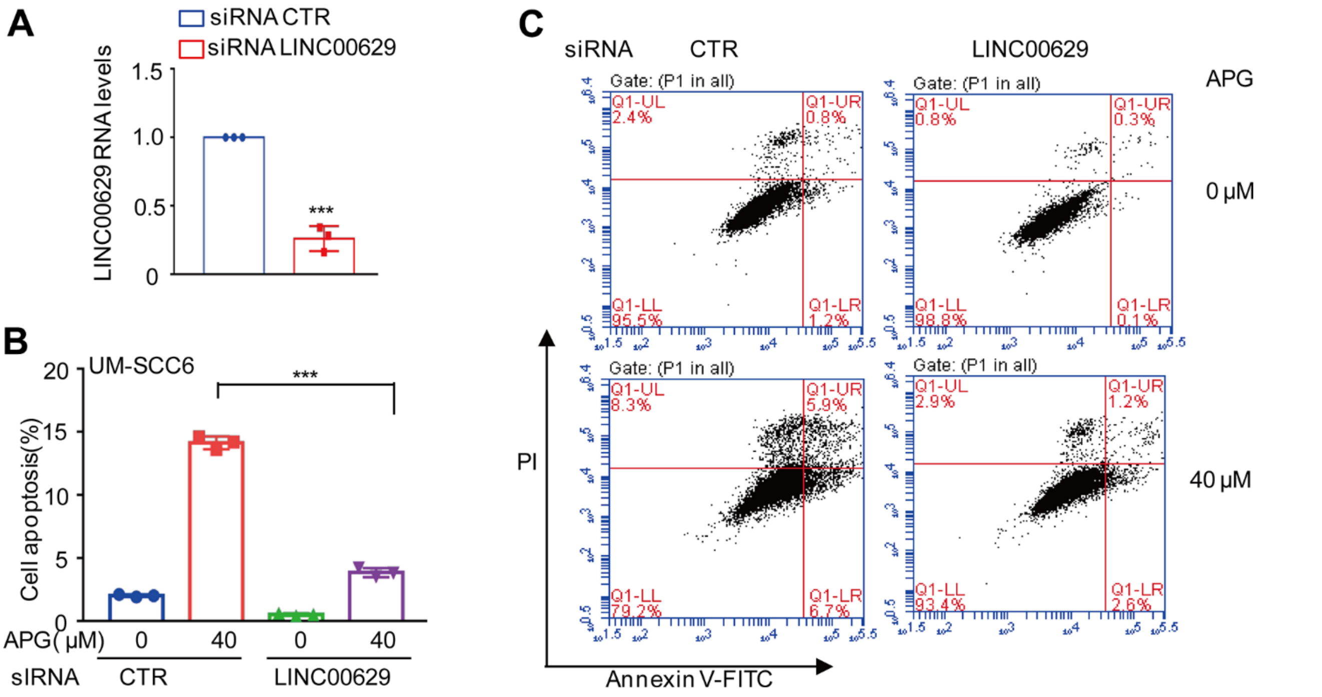

Supplementary Figure 2. (**A**) LINC00629 was knocked down in UM-SCC6 cells using siRNAs. The expression level of LINC00629 was determined by qRT–PCR. (**B**, **C**) Apoptosis was analyzed by flow cytometry. In (**A**, **B**) the results represent three independent experiments; *p<0.05, **p<0.01, ***p<0.001.

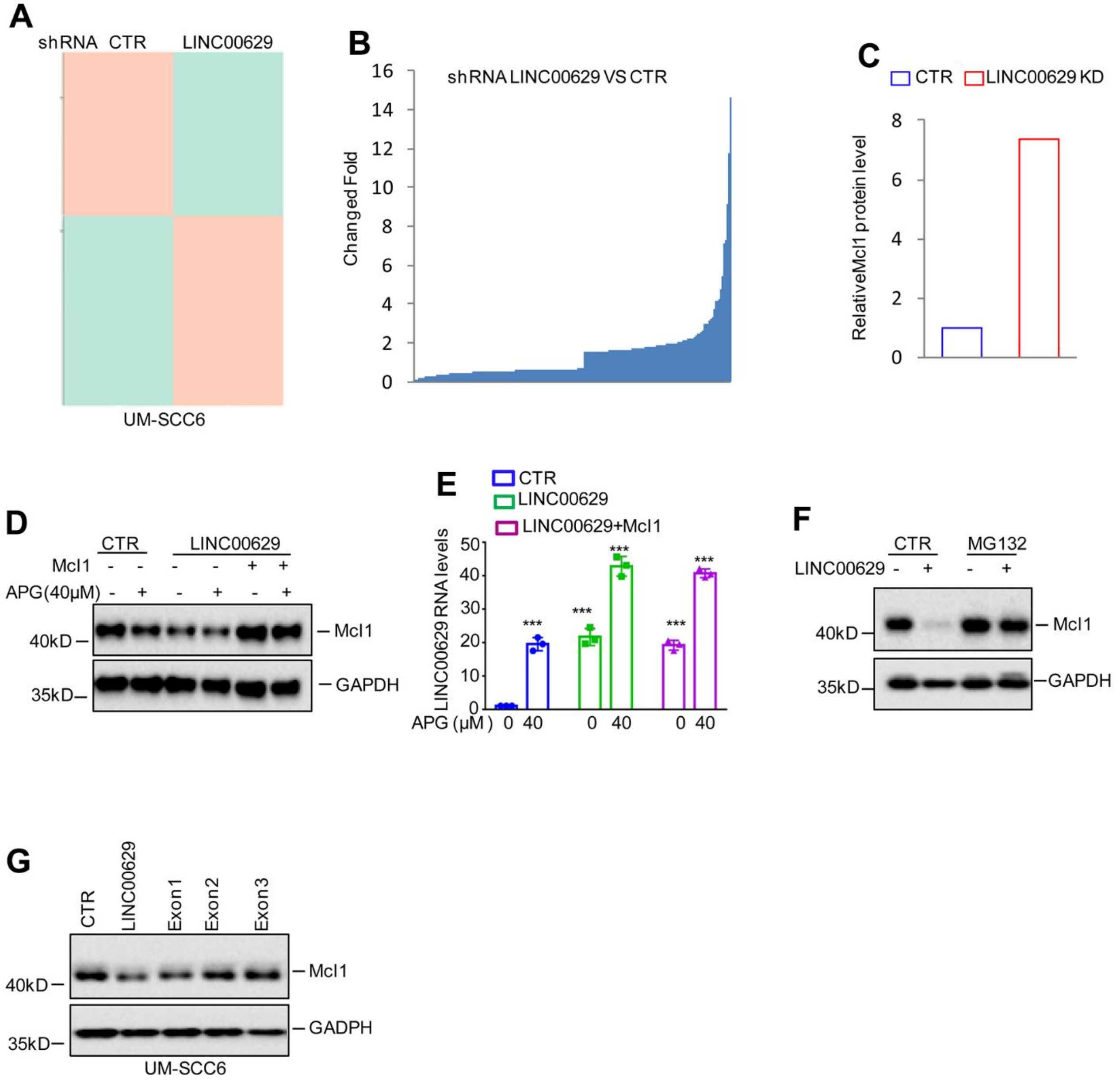

Supplementary Figure 3. (**A**, **B**) Differentially expressed proteins were identified by label-free quantitative proteomics in UM-SCC6 cells with or without LINC00629 knockdown. (**C**) The fold change in Mcl1 expression is shown. (**D**, **E**) Mcl1 was transfected into UM-SCC6 cells with or without LINC00629 overexpression, and the cells were then treated with apigenin as indicated. The expression levels of Mcl1 and LINC00629 were analyzed by Western blot and qRT-PCR. (**F**) LINC00629 was transfected into UM-SCC6 cells and then the cells were treated with MG132. The expression of Mcl1 was detected by Western blot. (**G**) The full length of LINC00629 and three truncated bodies were transfected into UM-SCC6 cell. The expression of Mcl1 was analyzed. In (**E**) the results represent three independent experiments; *p<0.05, **p<0.01, ***p<0.001.
