# Supplementary material for: LINC00629, a KLF10-responsive lncRNA, promotes the anticancer effects of apigenin by decreasing Mcl1 stability in oral squamous cell carcinoma
Source: Aging (Albany NY). 2022 Nov 28;14(22):9149–66. doi: 10.18632/aging.204396 (PMC9740369; doi:10.18632/aging.204396)
Supplement: Supplementary Table 1 [file aging-14-204396-s002.pdf]

SUPPLEMENTARY TABLE

Supplementary Table 1. The primers used in the article.

Gene	Forward primers 5'> 3'	Reverse primers 5'> 3'
LINC00629	AACCTGTTTATGCACCCTCC	AGTGTCCTCATAACATGGCAG
LINC00857	TCCGTTAAGCACCAGAAGTC	AGCAATATGGAAGGGAATGGAG
LINC00630	TGCAGTGAACATGGGAGTAC	AATCTGGCAAAGAGGGACTG
LINC01273	AGTCTCTGTTGCGGTGTTC	TGTTTTCCAGGTCCATAGGTG
LINC00342	TGCTGAGTAACTGTGATTCCC	CTTGGCTTCTCCCTCTAGTTG
SNHG8	GTCTACTCTGTCGCTCTTGTG	ACTTCGCCCATTACCACTTG
PTV1	CAAGTATTTTCTGAGCCTGATGG	ACAGCCTCCCTTAAAACCAC
LINC00511	CTCCACTTAGGCTTCACACTG	GCAACCTAGACCAATCAAACC
OCT4	GTGGAGGAAGCTGACAACAA	GCCGGTTACAGAACCACACT
Nanog	ACCTATGCCTGTGATTTGTGG	AGTGGGTTGTTTGCCTTTGG
CD133	GTGGATGCAGAACTTGACAAC	ACCCTTTTGATACCTGCTACG
Actin	ATCAAGATCATTGCTCCTCCTGAG	CTGCTTGCTGATCCACATCTG
KLF10	AAAGTTCCCATCTGAAGGCC	TCACAACCTTTCCAGCTACAG
KLF14	CACCAAAGCCTATTACAAGTCG	CAGTCGCAGGAGAAAGGG
NKX3-1	TCCCTGGTCTCCGTGTATAAC	TGCTTTTCATAGAGACACCCTG
FOXO6	ATCACCAAAGCCATCGAGAG	TGCTGTCGCCTTTATCCTTG
MEIS1	CACACCCTTACCCTTCTGAAG	TTGACTTACTGCTCGGTTGG
FOXA1	AGGGCTGGATGGTTGTATTG	TGAGTTCATGTTGCTGACCG
LBX	CGCCAGCAAGACGTTTAAG	GCCCAAAGATGGTCATACCG
LINC00629 Exon1	TGCCGGGACGGCGCAAGG	CCTGTCCCGGGGCCTCTC
LINC00629 Exon2	AAACCTGTTTATGCACCC	CTGAAGGGCAATGCAT
LINC00629 Exon3	CTGCCATGTTATGAGGAC	GCAAACATTACATGTTC
